# Cyberbullying and problematic internet use in adolescents with ADHD: exploring the relationship with moral disengagement and social skills

**DOI:** 10.3389/fpubh.2025.1577900

**Published:** 2025-06-03

**Authors:** Sigal Eden, Tali Heiman, Dorit Olenik-Shemesh, Yaacov B. Yablon

**Affiliations:** ^1^Faculty of Education, Bar-Ilan University, Ramat Gan, Israel; ^2^Department of Education and Psychology, The Open University, Ra'anana, Israel

**Keywords:** cyberbullying, PIU, social skills, moral disengagement, ADHD

## Abstract

This study explored the relationships between cyberbullying, problematic Internet use (PIU), moral disengagement, and social skills among children with and without Attention-Deficit/Hyperactivity Disorder (ADHD) due to the scarcity of research in this domain. The sample comprised 3,021 children aged 9–18 (*M* = 13.74; *SD* = 3.09), categorized into two groups: 2,247 (74.4%) typically developed (TD) children and 774 (25.6%) diagnosed with ADHD. Participants completed eight questionnaires assessing the study’s variables. Results revealed that children with ADHD displayed higher Internet use, greater PIU, and increased susceptibility to cyberbullying compared to TD peers, alongside elevated moral disengagement and lower social skills. Across both groups, heightened moral disengagement correlated with increased cyberbullying and PIU, while stronger social skills predicted reduced victimization, perpetration, and lower PIU. Moral disengagement emerged as a key factor influencing cyberbullying and PIU in both groups. These findings highlight the heightened risks for children with ADHD and provide insight for developing tailored interventions to address these challenges.

## 1 Introduction

As internet communication technologies evolve, they have become essential in daily life, especially for children and adolescents. However, intensive internet use has led to rising concerns about cyberbullying (54, 55), defined as deliberate and hostile behavior using electronic technologies to harass, embarrass, or intimidate others (1). While this definition tends to highlight the distinction between cyberbullying and traditional bullying, it may overlook the interconnection between the two forms, as research indicates that face-to-face school-based bullying and cyberbullying often overlap (2). Core elements such as repetition, power imbalance, intent to harm, and lack of justification can be present in both types of bullying (2). Nonetheless, cyberbullying possesses unique characteristics—for example, it can reach a broader audience, allows perpetrators to remain anonymous, and enables aggression to occur at a physical distance, which may reduce the perpetrator’s awareness of the harm inflicted (3–5).

Cyberbullying involves three roles: perpetrator, victim, and bystander (6) and has become increasingly prevalent among children, particularly in the post-COVID era (7). A recent review of longitudinal studies found cyberbullying rates among children ranging from 5.3 to 66.2%, with cybervictimization ranging from 1.9 to 84.0% (8). The escalating prevalence of cyberbullying is correlated to adverse outcomes including depression, anxiety, sleep disorders, and suicidal tendencies (9–11).

An additional adverse consequence of Internet utilization is problematic Internet use (PIU), characterized by an inability to regulate one’s Internet use, resulting in negative outcomes (12). Research shows a significant correlation between PIU and psychological stress in children (13). Moreover, PIU is a contributing factor to cyberbullying perpetration (14, 15), and a substantial predictor, with longer Internet usage linked to engagement in cyberbullying behavior (16). Extensive and problematic use of social media may further expose children to online aggression, including cyberbullying (17). Additionally, PIU has been associated with loneliness and depression while negatively impacting resilience, self-control, and school engagement, with girls showing higher susceptibility to PIU than boys (18).

Given the negative outcomes associated with PIU and cyberbullying, it is crucial to explore these behaviors in populations more vulnerable to self-regulation difficulties, such as individuals with Attention-Deficit/Hyperactivity Disorder (ADHD). The present study concentrates on children diagnosed with ADHD, a prevalent neurodevelopmental condition characterized by elevated levels of inattention, disorganization, and/or hyperactivity-impulsivity that surpass age-appropriate or developmental norms (19). These symptoms result in a reduction or impairment of social, behavioral, academic, and occupational functioning (20, 21, 56). ADHD affects approximately 3.5–5% of students globally within the educational system, with a fourfold higher incidence in males than females (19). Recent research indicates a worldwide surge in the prevalence rates of ADHD (57), underscoring the importance of considering these cases within the context of cyberbullying.

The distinctive challenges encountered by children with ADHD render them more susceptible to problematic behaviors and their repercussions, placing those children at an elevated risk of involvement in problematic technological phenomena, such as cyberbullying and PIU (22). Despite using technology in ways similar to the general population, children with ADHD exhibit an increased vulnerability to cyberbullying behaviors, both as victims and perpetrators, compared to their TD peers (6, 23, 58). Additionally, children with ADHD demonstrate higher instances of addictive Facebook and Internet use, along with elevated rates of PIU (23, 24). For instance, Morita et al. (59) identified relationships between PIU, hyperactivity/inattention, and depressive symptoms. This pattern extends into adulthood, with significant correlations observed between ADHD symptoms and PIU in adults with ADHD (60). Regrettably, there is a scarcity of dedicated studies examining the intersection of ADHD, cyberbullying, and PIU, as well as moral issues such as moral disengagement among adolescents, underscoring the heightened significance of our study.

Another significant challenge faced by children with ADHD is the development of social skills. Social skills refer to a set of abilities that facilitate individuals to interact and communicate with others. In practical terms, children and adolescents who exhibit strong social skills are typically characterized by confidence, self-awareness, self-control, empathy, cooperation, and self-sufficiency (25). In fact, a review of structural and functional magnetic resonance imaging (s/fMRI) literature on ADHD and disruptive behavioral disorders demonstrated an association with aggressive behavior and impulsivity (26).

In the absence of early adversities, TD children usually maintain stable social skills, a continuity that extends into adolescence and adulthood (27). However, children with ADHD sometimes experience significant social deficiencies that detrimentally affect the development of their social skills (28). This hindrance may potentially impede their ability to form positive personal connections with family, friends, acquaintances, and romantic partners (61).

Due to the challenges in cultivating robust social skills, children with ADHD often face exclusion by their peers during the crucial process of identity formation, resulting in fewer friendships (29, 30). Furthermore, research findings indicate that some children with ADHD exhibit aggressive behaviors toward both peers and authority figures, engage in disruptive conduct, cause damage to property, disrupt conversations, experience heightened frustration in play situations, and frequently violate rules (31).

Moreover, children with ADHD commonly encounter challenges in their relationships with parents, teachers, and peers due to difficulties in adhering to turn-taking norms, a tendency to engage in excessive talking, frequent interruptions, and restless fidgeting. These behaviors may contribute to their potential rejection by others, exacerbating the already existing difficulties in acquiring essential social skills, including those pertinent to online environments (32). In fact, there is a correlation between online risks, such as weak online connections, and offline risks, including diminished social skills (58). Internet addiction has been found to be negatively associated with peer relationships (62).

Previous research has established that Internet usage can diminish children’s social skills, with social phobia, prevalent among children with ADHD, predicting higher levels of PIU (63). Additionally, low self-esteem, social anxiety, and loneliness, commonly observed in this population, have been identified as predictors of cyberbullying victimization (64). ADHD and general hyperactivity in children have been identified as predictors of PIU (33, 65). Finally, antisocial behavior, emotional distress, and both hyperactivity and inattention have been demonstrated to sustain problematic technology use (34).

The association between deficits in social skills and both PIU and cyberbullying has also been identified in TD children (35–37).

Children encountering challenges in the development of appropriate social skills may eventually experience moral disengagement—a concept initially formulated by Bandura (38). Moral disengagement is a cognitive reconstruction process that enables individuals to dissociate from the constraints of their moral standards, permitting them to engage in unethical actions without experiencing distress or concern. This process involves the separation of moral reactions and the deactivation of self-condemnation mechanisms (66). As children progress beyond early childhood, they develop cognitive mechanisms that facilitate the activation of moral disengagement, allowing them to maintain a positive self-perception despite engaging in immoral behavior or actions (39).

There is a scarcity of studies specifically focusing on children with ADHD in the context of moral disengagement. However, a pilot study by Paciello et al. (40) found that children with ADHD and similar challenges are more prone to resorting to moral disengagement as a means of justifying immoral, aggressive, rule-breaking behaviors than their TD peers. Additionally, an examination of the association between individual differences in personality and aggressive behavior among children revealed a direct link between emotional instability, moral disengagement, and aggressive behavior (41).

As children transition into adolescence, the prevalence of moral disengagement may increase, influenced by environmental factors such as gaining permission to own a smartphone (42). This tendency could contribute to engaging in immoral behaviors such as PIU and cyberbullying. Research focused on children has consistently demonstrated a robust correlation between moral disengagement and both cyberbullying and problematic or compulsive Internet use (43–45). Notably, elevated levels of cyberbullying have been linked to a greater propensity for moral disengagement (46, 47), and high levels of moral disengagement via technology have been associated with both perpetration and victimization in the context of cyberbullying (48, 67).

Interestingly, the majority of studies in this domain have focused on TD populations, with a dearth of research exploring the correlation between cyberbullying or PIU and moral disengagement, specifically among children with ADHD.

## 2 Rationale and goals

Previous studies have consistently found negative effects of cyberbullying and PIU on the lives of children. It has also been found that children with ADHD are at higher risk for these negative effects, partly due to social difficulties. Although moral disengagement is recognized as a significant factor in the occurrence of cyberbullying and PIU, the precise nature of their relationship remains unclear, particularly in the context of children with ADHD. Moreover, research on moral disengagement among children with ADHD is limited, as this topic has received little prior attention. Therefore, the present in-depth study contributes to existing knowledge by exploring the social-psychological dimensions of cyberbullying and PIU, highlighting their associations with moral disengagement and social skills in a large sample of children with ADHD compared to their TD peers.

Consequently, Bandura’s (49) Social Cognitive Theory (SCT), provides a unified framework for understanding the interplay between cyberbullying, PIU, moral disengagement, and social skills, especially among children with ADHD. This model, known as reciprocal determinism, helps explain how individuals learn from observing others and how internal beliefs and self-regulatory processes shape behavior. SCT posits that behavior is influenced by a dynamic interaction between personal factors (e.g., cognitive processes such as moral disengagement, traits like ADHD), environmental factors (e.g., online settings), and behavioral patterns (e.g., cyberbullying, PIU). Within SCT, children learn moral standards from their environment, but may deactivate these standards using cognitive mechanisms (e.g., blaming the victim, minimizing harm). SCT also posits that social skills are learned through observation of others, reinforcement, and self-efficacy in social situations. Therefore, stronger social skills will lead to more empathic online interaction, particularly among children with ADHD, due to their deficits in executive functioning and emotion regulation.

Given the aforementioned evidence and the scarcity of pertinent research literature, this study aimed to investigate the correlation between cyberbullying and PIU and the variables of moral disengagement and social skills in a large cohort of TD children compared to children with ADHD.

Within this scope, several hypotheses were formulated:

- Hypothesis 1 (H1). Children with ADHD will show significantly higher levels of both cyberbullying and PIU compared to TD children.
- Hypothesis 2 (H2). Children with ADHD will show significantly higher levels of moral disengagement compared to TD children.
- Hypothesis 3 (H3). Children with ADHD will show significantly lower levels of social skills compared to TD children.
- Hypothesis 4 (H4). A correlation will exist between both cyberbullying and PIU, and moral disengagement and social skills. Specifically, higher levels of moral disengagement and lower social skills will predict increased instances of cyberbullying.

## 3 Materials and methods

### 3.1 Participants

Out of 3,625 children agreed to take part in the study, 80% completed the questionnaires. Therefore, the study comprised 3,021 Israeli children with an age range of 9–18 years (*M* = 13.74; *SD* = 3.09), including 1,309 boys (43.3%) and 1,712 girls (56.7%). All participants were randomly selected from three distinct Israeli school phases: primary school ages 9–12 (37.8%); middle school ages 13–15 (23.4%); and high school ages 16–18 (38.8%). The participants were categorized into two groups: (a) 2,247 TD children (74.4%); (b) 774 children with ADHD (25.6%), who received the diagnosis from either a psychiatrist or a neurologist. The information concerning the diagnosis was acquired through school-related avenues. Table 1 provides an overview of the participants’ descriptions.

**Table 1:** Sample description (*n* = 3,021).

Sample description	TD (*n* = 2,247)		ADHD (*n* = 774)		*X* ^2^
	*N*	%	*N*	%	
Grade					
Primary	831	72.7	312	27.3	3.66
Middle	542	76.7	165	23.3	
High school	874	74.6	297	25.4	
Gender					
Boys	913	69.7	396	30.3	26.00***
Girls	1,334	77.9	378	22.1	

*X2 stands for Chi-Square test results with three and one degrees of freedom, respectively.*

***p < 0.001.

Table 1 indicates a lower occurrence of ADHD in girls (22.1%) than in boys (30.3%), and this distinction was substantiated by the Chi-Square test (Chi = 26.00, *p* < 0.001). Conversely, the prevalence of ADHD among children at various grade levels was similar, ranging from 23.3% (middle school children) to 27.3% (primary school children).

### 3.2 Tools

- Demographic Questionnaire: This tool, comprising eight questions, pertains to background information about the children, encompassing factors such as gender, age, and ADHD diagnosis.
- Internet Frequency Use Questionnaire (7): This questionnaire, featuring 31 items, assesses adolescents’ Internet usage. Participants were instructed to evaluate the time they spent on various social platforms, including Facebook, TikTok, Instagram, Houseparty, etc. Items were rated on a 6-point Likert scale: 1 (*not at all*); 2 (*occasionally*); 3 (*1 h a day*); 4 (*2–4 h a day*); 5 (*5–7 h a day*); 6 (*8 + hours a day*). The item where the child reported the highest time allocation and use served as a representative measure for the frequency of Internet use. The aggregation was represented by the maximum time reported in one item, encompassing all other online activities. The internal consistency, as indicated by Cronbach’s alpha, was 0.92.
- Internet Skills [adapted from the Global Kids’ Online Survey; (68)]: This survey assessing children’s Internet skills consists of 17 items rated on a 5-point Likert scale ranging from 1 (*never*) to 5 (*always*). For example, “I can identify a message that appears suspicious to me.” The overall scale indicated a high internal consistency, with a Cronbach’s alpha of 0.96.
- Social Media Use Questionnaire [SMUQ; (50, 69)]: Previously employed in studies measuring PIU (70), this scale gauges problematic and excessive social media use. It consists of nine items, with two factors: withdrawal (5 items, e.g., “I struggle to stay in places where I will not be able to access the Internet”) and compulsion (4 items, e.g., “I lose track of time when I use the Internet”). Responses are rated on a 5-point Likert scale from 1 (*never*) to 5 (*always*). The total scale demonstrated good internal consistency, as indicated by a Cronbach’s alpha of 0.87 (Cronbach’s alpha of 0.83 for the withdrawal component and 0.82 for the compulsion component.
- Cyberbullying Questionnaire [(51), based on (4)]: Three out of six factors from the questionnaire were utilized, covering cyberbullies, victims, and bystanders. Participants assessed the frequency of bullying on different social platforms using a 5-point Likert scale, ranging from 1 (*never*) to 5 (*10 + times per month*). The victims’ scale demonstrated a Cronbach’s alpha of 0.79, the cyberbullying scale was 0.84, and the bystanders’ scale was 0.86.
- Prevention of Cyberbullying Survey (7): This survey, comprising 13 items, asked participants to note how frequently they acted to prevent or stop cyberbullying toward themselves as victims and toward others as bystanders. The items were on a 5-point Likert scale ranging from 1 (*never*) to 5 (*10 + times per month*). For instance, “I asked them to quit harassing me online.”
- Social Skills Rating Systems [SSRS-C; (71)]: This self-rating questionnaire includes 34 items intended to assess the child’s social skills. It comprises four social behavior subscales (cooperation, assertion, empathy, and self-control). For example, “I make friends easily.” Children graded each item on a 5-point scale, ranging from 1 (*never*) to 5 (*always*). The Cronbach’s alpha ranged from 0.82 to 0.91.
- Moral Disengagement in Bullying Scale [MDBS; (52)]: This scale measures adolescents’ moral disengagement from bullying situations. Children rated each of the 18 items on a 5-point scale, ranging from 1 (*disagree*) to 5 (*agree*). For example, “It’s okay to hurt another person a couple of times a week if you do it to protect your friends.” The Cronbach’s alpha was 0.84.

### 3.3 Procedure

Following approval from the University Research Ethics Board (10561), the Ministry of Education, teachers, and parents, participants from randomly diverse regions across Israel completed self-report questionnaires. Research assistants were present in classrooms to explain the procedure and answer any questions. The children then received an online link via Qualtrics and completed the questionnaires using computers or mobile phones. The survey process ensured confidentiality, with each participant providing responses confidentially. Only fully completed questionnaires were included in the analysis, and the response rate was notably high, reaching 80%.

### 3.4 Statistical methods

We employed the Generalized Linear Modeling (GLM) procedure to assess time differences in cyberbullying, PIU, moral disengagement, and social skills. In instances of a more intricate design involving additional independent variables, we employed the Successful Events over Trials option, representing the proportion of violence-experience events over a given set of occasions (53). This approach aligned with the targeted outcome, which focused on the frequency of cyberbullying experiences reported by respondents over an optional list.

This modeling approach assumes a discrete distribution while generating predicted probabilities for experiences. These predicted marginal probabilities were then compared and ranked across factor categories, such as grades, from the lowest to the highest. If significant effects were identified, the ranking was denoted by Latin letters, starting with the letter “a” for the lowest sub-group mean, and so forth. Wald’s Chi-Square test was employed to ascertain the significance level of each tested parameter.

### 3.5 Outcome measures

Along with one continuous measure for overall PIU, we established five outcome measures for cyberbullying:

(1) victimization,
(2) perpetration,
(3) bystanders,
(4) preventing self-victimization,
(5) preventing victimization of others.

These measures were presumed to indicate a discrete distribution (in terms of times experienced), except for the continuous PIU measure. The calculation process involved two steps. Initially, owing to the low frequency of cyberbullying experiences, we converted them into a binary scale—coded as 1 if the respondent experienced that type of violence and 0 if not. Subsequently, these binary items were treated as proportion outcomes, representing the actual experiences divided by the total number of violence items.

## 4 Results

Table 2 presents marginal mean proportions for the aforementioned cyberbullying and PIU measures, along with measures of social skills and moral disengagement. Internal consistency measures (Cronbach’s Alpha) across items are also provided in Table 2. All research indicators were computed based on multiple relevant items, and the results were presented separately for the ADHD and TD groups. Appropriate comparative tests (Chi-Square; Wald’s test) were then applied.

**Table 2:** Research variable statistics.

Measures	TD			ADHD			*X* ^2^	# items	α
	*M*	SD	*N*	*M*	SD	*N*	Wald		
Internet use: frequency	4.43^a^	2.93	2,220	5.29^b^	2.94	758	48.74***	31	0.92
Internet use: skills	3.15	1.15	2,204	3.21	1.15	752	1.65	17	0.96
Moral disengagement	1.48^a^	0.54	2,157	1.57^b^	0.66	740	13.42***	18	0.91
Social abilities	3.62^b^	0.58	1805	3.47^a^	0.63	604	28.98***	34	0.93
Cyberbullying: Perpetration	0.07^a^	0.002	1,649	0.12^b^	0.005	544	85.03***	8	0.90
Victimization	0.10^a^	0.003	1,648	0.17^b^	0.006	544	119.53***	8	0.92
Bystanders	0.19^a^	0.003	1,639	0.25^b^	0.007	537	78.75***	8	0.82
Preventing self-victimization	0.22^a^	0.003	1,648	0.30^b^	0.006	543	168.03***	12	0.92
Preventing victimization of others	0.22^a^	0.003	1,638	0.28^b^	0.006	537	81.76***	12	0.88
PIU	2.62^a^	0.94	2,197	2.81^b^	0.92	746	22.45***	9	0.89

α stands for Cronbach’s alpha index; Latin letters for marginal mean ranking from the lowest (“a”) and upward based on a multiple pairwise comparisons; Wald’s *X*^2^ stands for Wald’s test with one degree of freedom based on GLM results.

***p < 0.001.

As shown in Table 2, there are variations across all variables between the two research groups. The ADHD group exhibited more frequent Internet use and greater exposure to cyberbullying and PIU than the TD group, confirming hypothesis 1. Moreover, children with ADHD demonstrated higher levels of moral disengagement, confirming hypothesis 2, while TD children displayed higher levels of social skills confirming hypothesis 3. It is worth noting that the overall moral disengagement score was utilized in subsequent analyses due to the suboptimal performance of the moral disengagement subscales, falling below the accepted threshold of fit indices.

As previously mentioned, we employed the GLM procedure for continuous, proportion (events over trials), or binary response outcomes to examine potential determinants of PIU and cyberbullying from various perspectives. The GLM results are detailed in Tables 3–5.

**Table 3:** GLM results for internet use and skills, social skills, and moral disengagement across school level, gender, and ADHD.

Research variables	Internet frequency	Internet skills	Moral disengagement	Social skills
School level – Wald	248.89***	810.13***	43.80***	1.79
Primary	3.82^a^ (0.09)	2.52^a^ (0.03)	1.46^a^ (0.02)	3.52 (0.02)
Middle	5.21^b^ (0.11)	3.32^b^ (0.04)	1.65^c^ (0.02)	3.57 (0.03)
High	5.63^c^ (0.09)	3.74^c^ (0.03)	1.54^b^ (0.02)	3.54 (0.02)
Gender – Wald	20.45***	0.52	96.08***	20.59***
Boys	4.65^a^ (0.08)	3.21 (0.03)	1.65^b^ (0.02)	3.49^a^ (0.02)
Girls	5.12^b^ (0.08)	3.18 (0.03)	1.44^a^ (0.02)	3.60^b^ (0.02)
Diagnosis – Wald	64.95***	3.88*	8.99**	23.83***
TD	4.41^a^ (0.06)	3.15^a^ (0.02)	1.51^a^ (0.01)	3.61^b^ (0.01)
ADHD	5.36^b^ (0.10)	3.24^b^ (0.04)	1.59^b^ (0.02)	3.47^a^ (0.02)
AICC				
*N*	2,978	2,956	2,897	2,409

Latin letters for marginal mean ranking from the lowest (“a”) and upward based on a multiple pairwise comparisons; Wald for Wald’s Chi-Square test; Standard errors in parentheses.

***p < 0.001, **p < 0.01, *p < 0.05.

**Table 4:** GLM event over trial results for cyberbullying and PIU across school level, gender, and ADHD.

Research variables	Victimization	Perpetration	Bystanders	Preventing self-victimization	Preventing victimization of others	PIU
Step 1 – Wald Test						
School level – Wald	8.69*	11.63**	1.72	28.38***	35.54***	59.98***
Gender – Wald	96.23***	113.91***	39.35***	13.68***	6.22*	29.51***
Diagnosis – Wald	99.19***	69.14***	68.38***	160.99***	79.31***	31.34***
TD	0.11^a^ (0.003)	0.07^a^ (0.002)	0.19^a^ (0.004)	0.23^a^ (0.003)	0.23^a^ (0.003)	2.62^a^ (0.03)
ADHD	0.17^b^ (0.006)	0.12^b^ (0.005)	0.25^b^ (0.007)	0.31^b^ (0.006)	0.28^b^ (0.006)	2.81^b^ (0.03)
AICC	8,786.19	7,449.78	10,248.02	17,221.63	15,520.68	7,873.34
*N*	2,192	2,193	2,176	2,191	2,175	2,943
Step 2 – coefficients and SE						
Internet: frequency	0.11*** (0.01)	0.11*** (0.01)	0.07*** (0.01)	0.08*** (0.01)	0.08*** (0.01)	0.09*** (0.01)
Internet: skills	−0.11*** (0.03)	−0.20*** (0.04)	0.05* (0.02)	−0.01 (0.02)	−0.03 (0.02)	0.28*** (0.02)
Moral disengagement	0.97*** (0.04)	1.34*** (0.04)	0.77*** (0.04)	0.43*** (0.04)	0.67*** (0.03)	0.22*** (0.03)
Social skills	−0.56*** (0.04)	−0.81*** (0.05)	−0.18*** (0.04)	0.01 (0.04)	0.03 (0.03)	−0.07* (0.03)
AICC	5,917.46	4,450.60	7,563.80	9,664.79	11,711.75	5,658.61
*N*	1745	1745	1739	1,558	1738	2,408

Standard errors of the coefficients/parameters in parentheses. AICC for corrected Akaike’s Information Criterion.

***p < 0.001, **p < 0.01, *p < 0.05.

**Table 5:** ADHD interaction analysis of cyberbullying and PIU.

Research variables	Perpetration	Victimization	Bystanders	Preventing self-victimization	Preventing victimization of others	PIU
Step 3 – Wald Test						
Internet: frequency * Diagnosis	0.004	0.31	3.82*	5.49*	2.11	1.53
TD	0.11*** (0.01)	0.11*** (0.01)	0.08*** (0.01)	0.10*** (0.01)	0.09*** (0.01)	0.09*** (0.01)
ADHD	0.12*** (0.02)	0.12*** (0.02)	0.05** (0.02)	0.08*** (0.01)	0.07*** (0.01)	0.08*** (0.01)
Internet: skills * Diagnosis	29.34***	11.13***	3.00~	20.66***	5.62*	2.11
TD	−0.33*** (0.04)	−0.17*** (0.04)	0.02 (0.03)	−0.13*** (0.02)	−0.06** (0.02)	0.28*** (0.02)
ADHD	0.12 (0.07)	0.07 (0.05)	0.13** (0.05)	0.06 (0.03)	0.06 (0.04)	0.26*** (0.03)
Moral disengagement * Diagnosis	1.86	0.29	7.62**	2.42	0.62	0.01
TD	1.41*** (0.06)	0.99*** (0.05)	0.71*** (0.04)	0.61*** (0.03)	0.66*** (0.03)	0.24*** (0.03)
ADHD	1.21*** (0.07)	0.93*** (0.07)	0.87*** (0.06)	0.64*** (0.05)	0.68*** (0.05)	0.27*** (0.05)
Social skills * Diagnosis	2.92	4.07*	2.44	7.94**	23.17***	0.03
TD	−0.87*** (0.06)	−0.63*** (0.05)	−0.21*** (0.04)	−0.17*** (0.03)	−0.06 (0.04)	−0.07* (0.03)
ADHD	−0.64*** (0.09)	−0.40*** (0.08)	−0.08 (0.07)	0.03 (0.05)	0.27*** (0.05)	−0.07 (0.05)
AICC	4,078.53	5,902.40	7,556.78	13,002.09	11,681.97	5,590.21
*N*		1745	1739	1744	1738	2,408

Standard errors of the coefficients/parameters in parentheses.

***p < 0.001, **p < 0.01, *p < 0.05.

Table 3 provides the GLM results for six distinct research variables. The GLM models assessed their consistency across school levels, gender, and, notably, ADHD versus TD adolescents. The objective was to scrutinize how potential explanatory indicators varied based on the diagnosis factor, prompting further interaction analyses to delve into these effects. Gender and grade factors were introduced as controls.

Table 3 indicates that children with ADHD exhibited higher frequency of Internet usage and Internet skills and were more morally disengaged (Wald = 64.95, *p* < 0.001; Wald = 3.88, *p* < 0.05; Wald = 8.99, *p* < 0.01; respectively), while scoring lower in social skills than TD children (Wald = 28.83, *p* < 0.001). This suggests that ADHD children were more engaged in online activities, including risky ones, and less involved in social activities. Regarding school level differences, the frequency of Internet use and skills was higher among high school children and lowest among primary school children (Wald = 248.89, *p* < 0.001, Wald = 810.13, *p* < 0.001; respectively). In contrast, moral disengagement differed from the other ranking patterns (Wald = 43.80, *p* < 0.001). Children with higher moral disengagement were found at the middle school level, while high school children exhibited lower moral disengagement than primary school adolescents, with primary school children having the lowest moral disengagement. Social skills levels did not differ by school level, as these skills remained similar. Girls showed higher levels of Internet use frequency (Wald = 20.45, *p* < 0.001) and social skills (Wald = 20.59, *p* < 0.001), while boys exhibited higher levels of moral disengagement (Wald = 96.08, *p* < 0.001).

The subsequent analytical step involved regressing the outcomes of cyberbullying and PIU. The diagnosis factors were regressed in the preliminary step, followed by social skills measurements in the second step, and interactions between the ADHD group and the former in the third step. Table 4 presents the GLM result.

Consistent with prior findings, Table 4 highlights that cyberbullying and PIU were notably higher among ADHD children than their TD peers. Given the more frequent Internet use by ADHD adolescents, they exhibited greater susceptibility to various forms of cyberbullying. The second step incorporated Internet and social skills measurements to elucidate cyberbullying (unstandardized coefficients). As was hypothesized, more frequent Internet use was linked to all forms of cyberbullying (perpetration: *b* = 0.11, *p* < 0.001; victimization: *b* = 0.11, *p* < 0.001; bystanders: *b* = 0.07, *p* < 0.001; preventing self-victimization: *b* = 0.08, *p* < 0.001; preventing victimization of others: *b* = 0.08, *p* < 0.001; PIU: *b* = 0.09, *p* < 0.001). Intriguingly, Internet skills were negatively associated with cyberbullying but not uniformly across all outcomes (perpetration: *b* = −0.20, *p* < 0.001; victimization: *b* = −0.11, *p* < 0.001; preventing self-victimization: *b* = −0.05, *p* < 0.05), while Internet skills were correlated with higher PIU (*b* = 0.28, *p* < 0.001).

Confirming hypothesis 4, children displaying greater moral disengagement reported higher levels of all types of cyberbullying (perpetration: *b* = 1.37, *p* < 0.001; victimization: *b* = 0.97, *p* < 0.001; bystanders: *b* = 0.77, *p* < 0.001; preventing self-victimization: b = 0.43, *p* < 0.01; preventing victimization of others: *b* = 0.67, *p* < 0.001; PIU: *b* = 0.22, *p* < 0.001. In contrast, social skills were associated with reduced victimization and perpetration (perpetration: *b* = −0.81; victimization: *b* = −0.56, *p* < 0.001; bystanders: *b* = −0.18, *p* < 0.001; PIU: *b* = −0.07, *p* < 0.05). Social skills, however, showed no association with both types of cyberbullying prevention.

Table 5 extends the primary effect analysis from Table 4 while incorporating a test of the ADHD interaction effect. In other words, to assess the impact of each indicator on the cyberbullying experience, the model was separately run for ADHD children and TD adolescents. The supplementary outcomes of the two distinct models are presented sequentially.

Table 5 indicates that in Step 3, various interaction effects emerged. The frequency of Internet use by diagnosis consistently indicated a positive impact on cyberbullying and PIU across all outcome measures. However, these effects were uniform for perpetration, victimization, preventing victimization of others, and PIU, as corroborated by similar coefficients. When this interaction effect was deemed significant, namely for preventing self-victimization and as bystanders (Wald = 5.49, *p* < 0.05; Wald = 3.82, *p* < 0.05; respectively), the differences in coefficients were slightly more pronounced (ADHD: *b* = 0.08, *p* < 0.001; *b* = 0.05, *p* < 0.01 versus TD: *b* = 0.10, *p* < 0.001; *b* = 0.08, *p* < 0.001; respectively). In other words, this positive association between frequency of Internet use and the outcome was somewhat diminished among ADHD adolescents.

The division by diagnosis of the Internet skills effect was more consistent. Interaction effects were evident in perpetration, victimization, preventing self-victimization, and preventing victimization of others (Wald = 11.13, *p* < 0.001; Wald = 29.34, *p* < 0.001; Wald = 20.66, *p* < 0.001; Wald = 5.62, *p* < 0.05; respectively). Internet skills among TD children indicated a negative association with these outcomes (*b* = −0.17, *p* < 0.001; *b* = −0.33, *p* < 0.001; *b* = −0.13, *p* < 0.001; *b* = −0.06, *p* < 0.01; respectively). However, no significant association was discerned among the ADHD group.

A similar effect of moral disengagement was indicated for ADHD versus TD adolescents, except in the bystanders’ outcome (Wald = 7.62, *p* < 0.01), which resulted in a stronger positive effect among ADHD children than TD children (*b* = 0.87, *p* < 0.001; *b* = 0.71, *p* < 0.001; respectively). Finally, Internet skills interactions surfaced in victimization and preventing victimization (of self and others; Wald = 4.07, *p* < 0.05; Wald = 7.94, *p* < 0.01; Wald = 23.17, *p* < 0.001; respectively). The negative association between social skills and victimization was more pronounced among TD than ADHD children (*b* = −0.63, *p* < 0.001; *b* = −0.40, *p* < 0.001; respectively). Similarly, this difference persisted in perpetration (TD: *b* = −0.87, *p* < 0.001; ADHD: *b* = −0.64, *p* < 0.001). However, in preventing victimization of others, among ADHD adolescents, this association was positive (*b* = 0.27, *p* < 0.001), whereas among TD adolescents, it was deemed insignificant.

## 5 Discussion

The present study delved into the social-psychological dimensions of cyberbullying and PIU, emphasizing their correlation with moral disengagement and social skills in TD children versus children with ADHD. One of the innovations of the current study is the in-depth examination of these four important variables that have not previously been studied together, particularly in the comparison between children with and without ADHD. This in-depth investigation involved the use of eight research instruments that thoroughly assessed these aspects. Another strength of the study lies in its large sample size of 3,021 children, including 25.6% with ADHD.

Consistent with Hypothesis 1 (H1), our findings indicate that children with ADHD exhibit a heightened susceptibility to cyberbullying and PIU compared to their TD counterparts. While cyberbullying is recognized as an escalating issue among TD children (54, 55), the distinctive characteristics and challenges faced by children with ADHD appear to place them at an elevated risk for both PIU and cyberbullying, assuming roles of victims and perpetrators alike (e.g., 6, 22–24, 58). Their susceptibility to problematic behaviors is attributed to heightened levels of inattention, disorganization, and impulsivity, factors that may contribute to diminished social functioning (21, 56). Consequently, these impairments are anticipated to impact their online behaviors, culminating in elevated instances of cyberbullying and PIU.

It is noteworthy that, in the current study, children with ADHD were observed to engage in more frequent Internet use than their TD peers. This heightened Internet activity could potentially explain the increased instances of cyberbullying and PIU within the ADHD group. However, it is crucial to acknowledge that these children also demonstrated elevated Internet skills. Hence, it may be insufficient to attribute the heightened cyberbullying and PIU solely to more frequent Internet use. When examining these variables across the entire sample, irrespective of the two research groups, a consistent pattern emerged, revealing that more frequent Internet use was linked to various forms of cyberbullying (perpetration, victimization, bystanders, and preventing self−/other-victimization) and PIU. Surprisingly, while Internet skills were associated with reduced cyberbullying, they did not act as a deterrent for PIU and, in fact, seemed to enhance it. This underscores a nuanced conclusion regarding the role of Internet skills—mitigating cyberbullying but simultaneously contributing to elevated PIU.

Another contributing factor to the observed group differences may be rooted in social-psychological aspects, given that children with ADHD exhibit significant social impairments, struggle with interpersonal connections, and are frequently marginalized by their peers. These challenges manifest in disruptive behaviors and rule violations (28, 30, 31, 61). As mentioned, research on moral disengagement among children with ADHD is scarce, making the contribution of the current study most important. The linkage between emotional instability and moral disengagement leading to aggressive behavior (41) implies that children with ADHD may be more inclined to justify immoral actions through moral disengagement than their TD counterparts, a trend supported by a limited number of prior studies (26, 40, 41). Consequently, our hypotheses regarding the differences in moral disengagement (H2) and social skills (H3) between the two populations were substantiated, revealing that children with ADHD exhibited higher levels of moral disengagement, while TD children demonstrated superior social skills.

The core innovation of this study lies in marked the inaugural attempt to explore the relationship between cyberbullying, PIU, moral disengagement, and social skills in children with ADHD compared to their TD counterparts, as these four important variables have not previously been examined together, particularly in the comparison between children with and without ADHD. Confirming our final hypothesis (H4), heightened levels of moral disengagement were associated with increased cyberbullying and PIU across both ADHD and TD groups. Additionally, superior social skills were linked to reduced instances of cyberbullying and PIU, with stronger associations observed in the TD group regarding victimization and perpetration.

Building on Dawson et al.’s (58) insights into the correlation between online risks and diminished social skills, our findings align with prior research establishing connections between lower social skills and PIU, cyberbullying, and Internet addiction (35, 37, 62). While existing studies have explored the relationship between moral disengagement and cyberbullying or PIU (43–45), highlighting a positive association between high cyberbullying levels and increased moral disengagement (46), it is crucial to note that the majority of these studies have centered on TD populations. Notably, none have delved into the intricate relationship between cyberbullying, PIU, and moral disengagement in children with ADHD.

The social challenges faced by children, particularly the heightened risk of rejection experienced by those with ADHD, pose a significant hurdle in acquiring social skills relevant to online environments (32). Our findings indicate that compromised social skills, exacerbated by the prevalent low social skills observed in children with ADHD, as identified by Demirtaş et al. (63), contribute to heightened levels of PIU and cyberbullying victimization (64).

Consequently, CST (49) provides a joined framework for understanding the associations between all study’s variables—cyberbullying, PIU, moral disengagement, and social skills. As mentioned, this theory suggest that behavior is influenced by a dynamic interaction between personal, environmental, and behavioral factors. Hence, children with stronger social and moral skills are more likely to navigate online interactions with empathy and confidence, avoid becoming perpetrators or victims of cyberbullying, and use the Internet in a more regulated and constructive way.

The results of this study underscore the urgent necessity for implementing intervention and prevention strategies targeting cyberbullying and PIU among children with ADHD. Additionally, there is a need to explore approaches that can positively influence their social skills and moral disengagement.

### 5.1 Study limitations

While this study significantly advances our understanding of cyberbullying and PIU, and the correlation with moral disengagement and social skills, it is essential to acknowledge certain limitations. The reliance on self-report questionnaires introduces the potential for social desirability bias. To mitigate this concern, we took measures to guarantee the confidentiality and anonymity of participants, encouraging them to provide truthful responses.

Another limitation is that we did not examine the various subtypes of ADHD (e.g., inattention, impulsivity, and hyperactivity) in relation to the study’s variables, as well as the impact of medications. This should be addressed in future studies. Future research may also explore the moderating relationships among social skills and moral disengagement and cyberbullying and PIU. Moreover, several potential confounding variables could be assessed, such as anxiety and depression.

### 5.2 Conclusion

The findings of this study yielded interesting insights into the digital experiences of children with ADHD and underscore several key implications for enhancing their online well-being. Broadly speaking, individuals who use the Internet intensively demonstrate a heightened susceptibility to adverse consequences, including cyberbullying and PIU. Tentatively, it can be suggested that TD children utilize their Internet and social skills to mitigate cyberbullying, whereas ADHD children display a mixed impact of these skills on unfavorable outcomes. This may reflect underlying difficulties in emotional regulation, impulse control, and interpreting social cues—factors often associated with ADHD—that limit the effectiveness of their coping strategies in the online environment. Furthermore, moral disengagement emerged as an indicative factor for elevated cyberbullying and PIU across both groups, with potentially more pronounced effects among children with ADHD. This finding suggests that moral reasoning processes, particularly the ability to recognize and take responsibility for the impact of one’s online behavior, may be impaired or underdeveloped in this population. Consequently, interventions aimed at enhancing online well-being for children with ADHD should not only focus on improving social and communication skills but also explicitly address moral and ethical dimensions of digital behavior.

Our findings could serve as a basis for the development and evaluation of targeted, evidence-based interventions or support strategies tailored to the unique cognitive and behavioral profiles of children with ADHD to address their heightened susceptibility to cyberbullying and PIU. These might include:

- Structured programs that teach digital citizenship, emphasizing empathy, accountability, and safe online conduct.
- Social–emotional learning (SEL) interventions adapted for neurodiverse learners, with a focus on building resilience, perspective-taking, and self-regulation in online interactions.
- Parental and teacher training to help reinforce positive digital behaviors and recognize early signs of online distress or maladaptive use patterns.
- Ongoing monitoring and support mechanisms (e.g., digital mentors, peer support groups) that can provide real-time guidance in navigating online challenges.

Moreover, longitudinal studies could provide insights into the development of these issues over time and the potential impact of interventions on mitigating risk factors. Researchers may also find it valuable to delve deeper into the significant correlations revealed in our study within other at-risk populations.

In summary, the findings underscore the urgent need for multidimensional interventions that combine digital literacy, moral reasoning, and tailored social skills training to support the online well-being of children with ADHD. These efforts are essential not only to reduce exposure to harm but also to empower these children to engage more safely, ethically, and confidently in the digital world.

## Data Availability

The raw data supporting the conclusions of this article will be made available by the authors, without undue reservation.
